# Single-cell and spatial transcriptomics integration: new frontiers in tumor microenvironment and cellular communication

**DOI:** 10.3389/fimmu.2025.1649468

**Published:** 2025-10-02

**Authors:** Wenxin Shi, Zhiqiang Zhang, Xiaotong Xu, Yanpeng Tian, Li Feng, Xianghua Huang, Yanfang Du, Zhongkang Li

**Affiliations:** ^1^ Department of Obstetrics and Gynecology, The Second Hospital of Hebei Medical University, Shijiazhuang, China; ^2^ Hebei Key Laboratory of Regenerative Medicine of Obstetrics and Gynecology, Shijiazhuang, China; ^3^ Department of Obstetrics and Gynecology, Hebei General Hospital, Shijiazhuang, China; ^4^ Department of Obstetrics and Gynecology, The First Affiliated Hospital of Zhengzhou University, Zhengzhou, China; ^5^ Department of Obstetrics and Gynecology, The Fourth Hospital of Shijiazhuang, Shijiazhuang, China

**Keywords:** spatial transcriptomics, single-cell RNA sequencing, tumor microenvironment, cancer heterogeneity, intercellular communication

## Abstract

Single-cell RNA sequencing (scRNA-seq) has emerged as an advanced biological technology capable of resolving the complexity of cancer landscapes at single-cell resolution. Spatial transcriptomics(ST), as an innovative complementary approach, effectively compensates for the lack of spatial information inherent in scRNA-seq data. This review explores the rapidly evolving integration of scRNA-seq and ST and their transformative role in deciphering the tumor microenvironment (TME). We highlight how these technologies jointly uncover cellular heterogeneity, stromal-immune interactions, and spatial niches driving tumor progression and therapy resistance. Moving beyond previous reviews, we emphasize emerging computational strategies for data integration—including deconvolution and mapping approaches—and evaluate their applications in characterizing immune evasion, fibroblast diversity, and cell-cell communication networks. Ultimately, this review provides a forward-looking perspective on how spatial multi-omics are poised to advance precision oncology through spatially-informed biomarkers and diagnostic tools. We conclude that the full clinical potential of these technologies relies on closing the gap between analytical innovation and robust clinical implementation.

## Graphical Abstract

The application of single-cell RNA sequencing (scRNA-seq) and spatial transcriptomic (ST) techniques in TME.

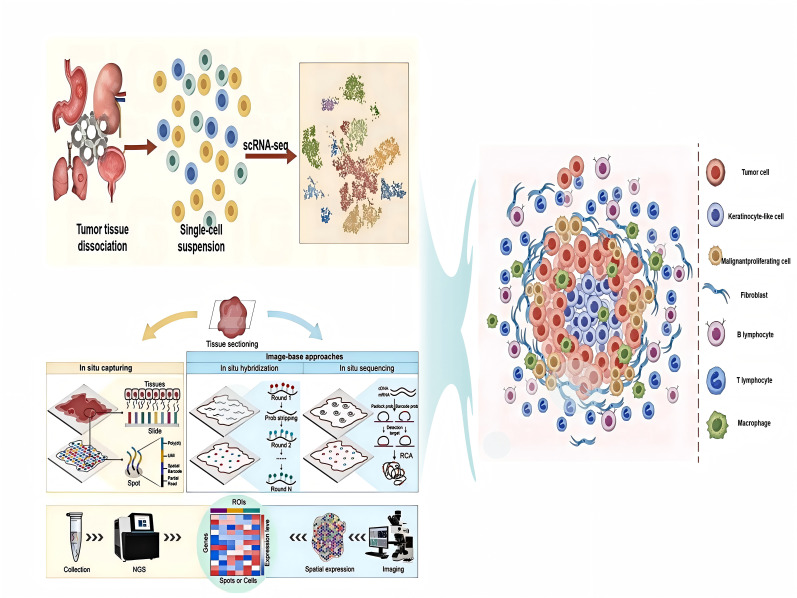

## 1 Introduction

Traditionally, tumors have been regarded as diseases primarily characterized by uncontrolled proliferation of malignant cells, and therapeutic strategies have predominantly focused on their direct eradication through chemotherapy and radiotherapy. However, this perspective has evolved significantly with the growing recognition of tumor complexity, wherein tumors are increasingly viewed as highly dynamic and heterogeneous ecosystems (1). The TME, in particular, represents a complex cellular and molecular landscape composed not only of malignant cells but also of diverse non-malignant components, including immune cells, cancer-associated fibroblasts (CAFs), vascular endothelial cells, pericytes, and tissue-resident stromal cells, all embedded within the extracellular matrix (ECM) (2). In certain tumor types, non-malignant cells may constitute the majority of the tumor mass (3). The cellular composition and functional states in the TME exhibit significant variability influenced by factors such as the anatomical origin of the tumor, genetic and epigenetic features of cancer cells, disease stage, and host-specific factors (4–6). Understanding the complex cellular interactions and spatial heterogeneity in the TME is crucial for enhancing our understanding comprehension of tumor biology and facilitating the development of more precise and effective anticancer therapies.

Despite its central role in cancer progression and therapeutic response, the TME presents significant analytical challenges. One primary limitation arises from the technical constraints of transcriptomic profiling methods. Conventional bulk RNA sequencing (RNA-seq) captures only average gene expression from heterogeneous cell populations, thereby obscuring intrinsic cellular heterogeneity in the TME and failing to identify rare but functionally critical subpopulations (7, 8). Tumor heterogeneity itself constitutes another substantial barrier (9–11). This heterogeneity exists both across patients (inter-tumor heterogeneity) and within individual tumors (intra-tumor heterogeneity), as cancer cells occupy various differentiation states while exhibiting divergent transcriptional profiles and mutational landscapes. Furthermore, non-malignant cell populations, including immune and stromal cells, exhibit extensive phenotypic and functional diversity. The complexity of mechanisms underlying therapy resistance further highlights the urgent need for deeper insights into the TME (12–15). Increasing evidence suggests that non-malignant cells actively contribute to resistance against chemotherapy, targeted therapies, and immunotherapies through multiple mechanisms. For instance, CAFs secrete ECM components and growth factors, establishing physical and biochemical barriers that hinder drug penetration (16, 17). Immunosuppressive cells such as regulatory T cells (Tregs) and M2-polarized macrophages suppress anti-tumor immunity by expressing immune checkpoint molecules (e.g., *PD-1*, *CTLA-4)* and releasing inhibitory cytokines such as *IL-10* and *TGF-β* (18–20). Collectively, these findings underscore the necessity of comprehensively characterizing the TME—encompassing cellular composition, functional phenotypes, and spatial interaction networks—to inform the rational design of combination therapies (21, 22). To address these challenges, the integrating scRNA-seq with ST has emerged as a powerful strategy. This approach facilitates insights into the spatial and functional complexity of the TME.

scRNA-seq is a powerful technique enabling high-resolution gene expression profiling for the individual-cell level, enabling the identification and characterization of distinct cellular subpopulations with specialized functions (23). ST, a rapidly evolving complementary approach, maps gene expression within intact tissue sections, preserving critical spatial context and tissue architecture (24). Given the cellular complexity of the TME, no single technology can fully capture its spatial and functional heterogeneity. Although current ST platforms generally lack true single-cell resolution, their integration with scRNA-seq provides a comprehensive perspective on the TME. Combining scRNA-seq and ST overcomes these limitations by bridging cellular identity with spatial localization. For instance, multimodal intersection analysis (MIA) was introduced in 2020 to integrate scRNA-seq and ST data, aiming to map spatial associations cell-type relationships in pancreatic ductal adenocarcinoma (PDAC) (25). This study revealed that stress-associated cancer cells colocalize with inflammatory fibroblasts, the latter identified as major producers of interleukin-6 (*IL-6*), underscoring spatially organized tumor-stroma crosstalk in PDAC (25).

The integration of scRNA-seq and ST enables researchers to dissect the complexity and spatial organization of the TME with unprecedented resolution. This synergistic approach not only deepens our understanding of tumor biology but also accelerates the discovery of novel diagnostic and prognostic biomarkers, paving the way for more precise and effective therapeutic strategies. We conducted comprehensive searches in PubMed, Web of Science, and Scopus to ensure broad coverage of relevant studies. We used a combination of keywords related to single-cell sequencing, spatial transcriptomics, tumor microenvironment, cancer heterogeneity, and their respective applications in oncology. We defined explicit criteria for including studies based on relevance, study type (e.g., original research, key reviews), and publication status. Studies were excluded if they were not peer-reviewed, not published in English, or deemed outside the scope of this review. We focused primarily on literature published between January 2010 and June 2025 to capture the most recent and impactful advances in the field. (GA).

## 2 Advances in technologies for analyzing spatial distributions

ST is an emerging technology that enables spatially resolved gene expression profiling within intact tissue sections, preserving the native histological context. By combining high-resolution imaging and transcriptomic analysis, ST maps gene expression patterns with precise spatial localization, achieving subcellular resolution in some cases. Current ST methodologies can be broadly classified into two categories: image-based (I-B) and barcode-based (B-B) approaches (26, 27). Image-based methods, such as *in situ* hybridization (ISH) (28) and *in situ* sequencing (ISS) (29), utilize fluorescently labeled probes to directly detect RNA transcripts within tissues, allowing visualization of gene expression patterns while maintaining spatial integrity. In contrast, barcode-based approaches rely on spatially encoded oligonucleotide barcodes to capture RNA transcripts. In solid-phase transcriptome capture, RNAs hybridize to immobilized barcoded probes on slides before sequencing. Deterministic spatial barcoding assigns unique barcodes to each transcript, retaining positional information throughout sequencing (30–32). These complementary strategies facilitate comprehensive spatial transcriptome profiling, when integrated with single-cell techniques, they yield unprecedented resolution for investigating tissue architecture and tumor heterogeneity.

scRNA-seq of patient-derived tumors has uncovered diverse cellular subpopulations and revealed intricate intercellular communication networks within the TME (33–39). However, scRNA-seq requires tissue dissociation, leading to the loss of spatial context and limiting insights into tissue architecture and cell-cell interactions. To address this, several strategies have been developed to preserve or reconstruct spatial information. For example, combining ISH-based gene expression mapping with scRNA-seq data has proven effective for identifying rare cell types and subpopulations using targeted gene panels (40, 41). Recent advances have evolved ISH into high-plex RNA imaging (HPRI) techniques, including *in situ* sequencing, multiplexed error-robust fluorescence *in situ* hybridization (MERFISH) (42), and sequential fluorescence *in situ* hybridization (seqFISH) (43–45). However, these approaches are often limited to well-defined tissues and remain challenging when applied to heterogeneous solid tumors characterized with complex structures and diverse transcriptomic profiles.

Emerging methods, such as sci-Space, have been developed to address this limitation by generating spatially resolved transcriptomic maps at near-single-cell resolution across extensive tissue areas. In mouse embryonic development studies, sci-Space enabled the simultaneous capture of approximate spatial coordinates and complete transcriptomes from over 120,000 nuclei. However, its spatial resolution is currently limited to approximately 200 micrometers. Although there have been improvements in spot density and size, the resolution remains insufficient for precisely capturing interactions between neighboring cells. As a result, this approach typically yields composite transcriptomic profiles derived from small cell clusters or cellular fragments rather than genuine single-cell resolution. Currently, ST remains one of the most widely adopted approaches for high-throughput spatial gene expression analysis (46, 47). scRNA-seq is a high-throughput method for transcriptomic profiling at individual-cell resolution. By isolating individual cells, capturing their mRNA, and performing high-throughput sequencing, scRNA-seq reveals cellular heterogeneity typically masked in bulk RNA analyses. The advantages of scRNA-seq include: (i) identification of rare cell populations, including tumor stem cells and transitional cellular states, which are undetectable by bulk RNA-seq (48); (ii) classification of cells based on canonical markers, enabling precise identification of immune cell subsets and epithelial cell states (49); (iii) characterization of dynamic biological processes, such as differentiation trajectories and cellular transitions (50); and (iv) integration with multi-omics approaches, including single-cell ATAC-seq (chromatin accessibility) and CITE-seq (surface protein expression), providing multidimensional insights into cell states (51).

Despite these strengths, scRNA-seq also exhibits notable limitations. RNA capture efficiency per cell is relatively low (52). The method remains costly and technically challenging, necessitating careful optimization of sample processing protocols (53, 54). Critically, the mandatory tissue dissociation disrupts native spatial relationships, hindering analysis of cell–cell interactions within intact tissue architectures (55, 56). The comparison of scRNA-seq and ST is shown in **Table 1**.

**Table 1:** Comparison between scRNA-seq and ST technology.

Characteristic	scRNA-seq	Spatial transcriptome
Resolution	Single-cell level	spot level (multiple cells)
Spatial information	Missing	Retain
Organizational handling	Dissociate into single cells	Tissue section
Advantage	Fine identification of cell types	Spatial relationship retention
Limitations	Lost spatial background	Limited resolution
Application scenarios	Cell atlas construction and rare cell identification	Spatial niche analysis, cell interaction

The integration of scRNA-seq and ST confers significant advantages for deciphering complex biological systems: (i) Comprehensive gene expression profiling: scRNA-seq enables high-resolution gene expression analyses, revealing cellular heterogeneity and transcriptional dynamics within tissues (8). It is essential for cell-type identification, developmental tracking, and elucidating disease mechanisms. (ii) Spatial context and tissue architecture: ST preserve native tissue spatial architecture, enabling localization of gene expression patterns, cellular distributions, and intercellular interactions (57). (iii) Complementary strengths: While scRNA-seq lacks spatial information, ST technologies face resolution and throughput limitations. Their integration overcomes their individual limitations, offering a comprehensive understanding of tissue biology (58–60).

Combining scRNA-seq and ST provides deeper insights into cellular interactions with their microenvironment (61–63), with critical implications for both diagnostics and therapeutics. This integrative strategy supports the identification of spatially informed biomarkers and therapeutic targets by linking gene expression patterns to precise tissue regions, thereby advancing personalized medicine and enhancing disease diagnosis (64). Currently, two major computational approaches are used to this integration: deconvolution and mapping. Deconvolution utilize single-cell reference datasets to computationally estimate the cellular composition within each spatial capture spot, determining proportions of various cell types. Mapping approaches assign scRNA-seq-defined cellular subtypes to cells within spatial maps or localize individual scRNA-seq profiles to specific tissue niches. The characteristics of different integration strategies are shown in **Table 2**. These analyses provide critical spatial context to inferred ligand–receptor interactions and other forms of intercellular communication derived from scRNA-seq data.

**Table 2:** seRNA-seq and spatial transcriptomic integration strategies.

Integration strategies	Methods	Advantages	Disadvantages	Re
Deconvolution	SPOTlight, CellPhoneDB	High accuracy	Does not incorporate capture location information when modeling spatial decomposition	(65)
Deconvolution	Cottrazm	Provide spatial quantitative information on cell composition	Highly dependent on the quality and completeness of reference data	(66)
Deconvolution	CARD	More precise	High computational complexity	(67)
Deconvolution	cell2location	Absolute quantification, not relative proportion	It has a high computational complexity and is extremely time-consuming	(68)
Deconvolution	cell2location	This provides strong and quantifiable evidence of spatial composition.	The technical deviation that cannot be completely avoided and lack the standard verification	(69)
Deconvolution	cell2location	Absolute quantification	Highly dependent on the quality and matching degree of reference data	(70)
Deconvolution	RCTD	Greatly enhance the detection sensitivity and deconvolution accuracy for target cell types, especially rare subtypes	RCTD will force the entire expression signal of each bin to be attributed to a combination of fibroblast subtypes	(71)
Deconvolution	SPOTlight	Higher resolution, capable of revealing cellular interactions	the high spatial heterogeneity among samples	(72)
Deconvolution	SPOTlight MIA	No external reference data is required	It may confuse cell types and states	(73)
Deconvolution	cell2location	It can handle the inherent over-dispersion and technical noise in single-cell and spatial data very well, and the results are more robust and reliable	Biological verification is still required	(74)
Deconvolution	CARD MISTy	The functions complement each other perfectly, forming an analytical closed loop	The accuracy of MISTy analysis is highly dependent on the accuracy of RCTD deconvolution	(75)
Deconvolution	CARD	Hierarchical annotation strategy improves accuracy	The recognition ability is limited and it is unable to parse new cell states	(76)
Deconvolution	SPOTlight, CellTrek	Through multi-level and multi-angle verification, the conclusion is extremely robust	The analysis process is extremely complex and requires extremely high professional knowledge	(77)
Mapping	Tangram	Compatible with capture and image-based ST data	Gene expression can be less accurately predicted from histology images if the cells cannot be segmented	(59)
Mapping	CellTrek	Capture the complex nonlinear relationship between gene expression and spatial position	The spatial position of cells is predicted by the model rather than directly measured through experiments	(78)
Mapping	CellTrek	Realize spatial mapping at the single-cell level	high requirement for data matching degree	(79)
Mapping	CellTrek	true single-cell resolution spatial mapping	It is required that the scRNA-seq data and ST data must be derived from highly similar biological backgrounds	(80)
Spatially informed ligand–receptor analysis	SpaOTsc	The majority of cells can be mapped accurately using a small number of genes.	gnores the possible time delay associated with cell-to-cell communication	(81)

## 3 Immunosuppressive tumor microenvironment

The immunosuppressive tumor microenvironment (ITME) is a specialized ecosystem in tumor tissues. The ITME suppresses anti-tumor immune responses via multiple mechanisms. This promotes immune escape, tumor growth, and therapy resistance. Complex cellular crosstalk drives ITME formation, representing a major challenge to immunotherapy. The interaction between immune-mediated tumor editing and cancer cell immune evasion influences disease progression and therapeutic outcomes (82, 83).

### 3.1 Cells in the tumor microenvironment

The TME comprises diverse cell types that collectively influence tumor behavior. ScRNA-seq and ST have revealed unprecedented heterogeneity and functional plasticity among these populations, uncovering their roles in immune evasion, metastasis, and treatment resistance.

#### 3.1.1 CD8^+^ T cells

CD8^+^ T cells, also known as cytotoxic T lymphocytes (CTLs), serve as central effectors in anti-tumor immunity. They mediate tumor cell killing through cytolytic mechanisms, such as perforin and granzyme release, and secrete cytokines like *IFN-γ* to amplify immune responses. However, in the ITME, chronic antigen exposure, inhibitory signals, and metabolic disturbances often lead to CD8^+^T cell immune exhaustion (84–86). T cell exhaustion is a critical factor contributing to immune evasion and limited immunotherapy efficacy. Recent studies suggest that targeted transcriptional modulation (87), metabolic reprogramming (86, 88), and microenvironmental remodeling (89, 90) can restore CD8^+^ T cell functionality. These strategies offer promising directions for next-generation immunotherapies.

ST has become an essential tool for deciphering the functional states and spatial organization of CD8^+^ T cells in the TME. By mapping spatial proximity to other cell populations, ST can infer intercellular communication and elucidate how local cellular neighborhoods influence CD8^+^ T cell phenotypes (91–94).

#### 3.1.2 CD4^+^ T cells

CD4^+^ T cells act as central coordinators of immune responses and differentiate into various functional subsets. In the TME, their activity is highly context-dependent, influenced by subset composition, cytokines and metabolites. scRNA-seq has revealed that CD4^+^ T cells can exert tumor-suppressive effects by producing *TNF-α*, while ST indicates spatial co-localization with CD8^+^ T cells, suggesting coordinated immune responses. These findings exemplify the complementary strengths of integrating scRNA-seq and ST (95). Future research should leverage these technologies to explore CD4^+^ T cell heterogeneity and spatial organization, facilitating precision immunotherapy.

CD4^+^ T cells mediate anti-tumor effects through both indirect and direct mechanisms (95). Dynamic changes in CD4^+^ T cell subsets correlate with tumor progression. For instance, scRNA-seq analyses of prostate cancer identified elevated regulatory T cell (Treg) activity scores in tumors relative to normal tissue, with tumor-infiltrating Tregs displaying increased expression of TNF receptor family genes. These findings suggest CD4^+^ T cells may promote both pro-inflammatory tumor progression and immunosuppressive niche formation via TNF signaling (96).

#### 3.1.3 Tumor-associated macrophages

Macrophages represent essential innate immune components, mediating pathogen clearance and immune modulation. Within tumors, macrophages—termed tumor-associated macrophages (TAMs)—often exhibit immunosuppressive functions and promote tumor progression. TAMs exhibit remarkable plasticity, polarizing into pro-inflammatory, cytotoxic M1-like or immunosuppressive, tissue-remodeling M2-like phenotypes (97–99).

A recent study analyzed 97 paired samples from 24 colorectal cancer patients with liver metastases using scRNA-seq and spatial transcriptomics. It revealed extensive spatial remodeling in metastatic niches, driven largely by MRC1^+^CCL18^+^ M2-like macrophages (100). However, how the chemotherapy induces the functional changes of macrophages was not clear. Further experimental validation is required to validate that such state shift of macrophages is due to altered differentiation or population change. It showed intensified immunosuppression, highlighting the therapeutic potential of targeting M2-like TAMs (100). Similarly, a 2021 breast cancer study using scRNA-seq identified immunosuppressive macrophage subsets—lipid-associated macrophages (LAMs) and CXCL10^+^ macrophages—as key producers of suppressive cytokines. ST further demonstrated their proximity to PD-1^+^ lymphocytes (101). However, its number of cases per clinical subtype limited to estimate subtype-specific features.

In clear cell renal cell carcinoma (ccRCC), ST revealed distinct expression profiles between tumor cores and boundaries. Integrative analysis identified selective expression of *IL-1β* by macrophages at tumor edges. *IL-1β* expression correlated with epithelial–mesenchymal transition (EMT) induction and poor prognosis. *IL-1β* blockade reduced tumor burden in RCC murine models (102), while in another study, it was verified that *IL-6* lowered lung cancer incidence (103), highlighting *IL-1β* as a promising therapeutic target (104).The limit is that the researchers chose mouse renal cell carcinoma lines as the tumor cell model. This cell line usually lacks mutations related to ccRCC (103).

### 3.2 Tumor cell–immune cell communication in the tumor microenvironment

Communication between tumor cells and immune cells in the TME, critically influences immune evasion or tumor eradication. scRNA-seq approaches have elucidated cell–cell interaction networks and identified pivotal immune cell signaling hubs (103). By inferring ligand–receptor interactions from scRNA-seq data, researchers can delineate intercellular communication pathways between cancer and TME, including those driving immunosuppression (105). Notably, epithelial cells engage strongly with myeloid cells and may demonstrate potential immunosuppressive communications with T cells.

Cell-cell interactions within the tumor microenvironment drive key processes including immune suppression, angiogenesis, and metastasis. Advances in single-cell and spatial multi-omics now enable systematic mapping of these communications, revealing ligand–receptor networks and functional cellular crosstalk. Targeting these interactions offers promising strategies for novel cancer immunotherapies.

#### 3.2.1 T lymphocyte-cell interactions

Interactions between T lymphocytes and various tumor cells play a critical role in shaping the immune microenvironment. scRNA-seq analyses have revealed strong immunosuppression in tumors, characterized by increased infiltration of regulatory T cells (Tregs), which impair CD8^+^T cell cytotoxicity and promote tumor progression (106). ST further identified immune hotspots where Tregs are found in close proximity to effector T cells, suppressing anti-tumor responses within these regions (107, 108). Consistent with this, transcriptomic profiling shows elevated abundances of Tregs and exhausted CD8^+^T cells, underscoring the profound immunosuppression and immune infiltration features in the tumor microenvironment (109).

#### 3.2.2 TAM-cell interactions

In TNBC tumors, macrophage subsets often co-express both M1 and M2 markers, suggesting their dual role in either suppressing or promoting tumor progression and metastasis (110). Specific subpopulations of tumor-associated macrophages (TAMs) are associated with T cell infiltration and immunosuppression, highlighting their critical influence on the immune landscape of TNBC (111). These TAMs can impair T cell function and dampen immune responses, thereby supporting immune evasion and fostering a tumor-permissive microenvironment (112, 113). Interestingly, macrophage infiltration also correlates with improved patient outcomes. Transcriptome studies indicate that a high density of CD163^+^macrophages is significantly associated with longer overall survival and TNBC-specific survival (114).

#### 3.2.3 CAFs-cell interactions

It was showed that CAF phenotypes were a strong prognostic factor, and CAF phenotypes associated with good and poor patient prognosis. It was also discovered that different CAF types varied in their spatial distribution in the TME (**Table 3**). However, the interactions occurred at the edges of the cells was not investigated (115). In another study, the intercellular communication predominantly involved iCAFs, malignant epithelial cells, mCAFs, and pCAFs, each exhibiting distinct numbers and strengths of interactions. Although their study provided a detailed analysis of CAFs, it may not fully encapsulate all interactions and mechanisms (116). ST in breast cancer have revealed specific spatial enrichment between cancer-associated fibroblasts (CAFs) and T cell subsets (101). In multiple tumor types, certain CAF subsets are associated with T-cell exhaustion. For example, ecm-myCAF and TGF-β-myCAF in breast cancer, and a FAP^+^/PDGFRA^-^subset in lung cancer, have been linked to this immunosuppressive process (117, 118). Consistent with this, a separate lung cancer study also reported positive correlations between FAP^+^ CAFs and T-cell exhaustion markers (119). Spatial transcriptomics in head and neck cancer demonstrated co-localization of specific CAF subsets with exhausted T cells (120).

**Table 3:** Subtypes and comparisons of CAFs.

Subtype	Main gene	Function	Clinical significance
myCAF	ACTA2 (α-SMA), TAGLN, MYL9, CNN	High contractility, generating a large amount of ECM; It forms a physical barrier that hinders T cell infiltration and drug delivery; It is usually strongly activated by the TGF-β signaling pathway	It may be related to tumor hardness, invasion, metastasis and immune rejection
iCAF	IL6, LIF, CXCL12, CXCL1, CXCL2,	Secrete a large amount of cytokines and chemokines; Recruit myeloid cells and induce immunosuppression; Promote the stemness and survival of tumor cells; It is usually driven by the IL-1α/β and NF-κB signaling pathways.	It may be related to immunosuppression, inflammation and resistance to chemotherapy.
apCAF	CD74, MHC-II	It expresses MHC-II class molecules but lacks co-stimulatory molecule; It may mediate the impotence or inhibition of CD4+ T cells rather than their activation.	Unclear
meCAF	CAV1, ALDH1A	Metabolic reprogramming to support the metabolic needs of tumors; Nourish tumor cells through nutrients	It may be related to tumor growth, metabolic adaptation and treatment resistance.

#### 3.2.4 B lymphocyte-cell interactions

B cells influence the tumor microenvironment not only through antibody production, but also via cytokine secretion and direct cell-cell interactions. They exert regulatory effects on both tumor cells and other immune cells. For instance, ligand-receptor interactions can mediate direct contact between B cells and tumor cells (121). Such interactions may also suppress antibody-mediated immune responses (122). Together, these mechanisms help sustain an immunosuppressive microenvironment, promoting tumor proliferation and metastasis.

## 4 Functional heterogeneity of cancer-associated fibroblasts and their immunomodulatory roles

CAFs are a major stromal component in the TME, critically contributing to tumor initiation, progression, invasion, metastasis, and therapeutic resistance. CAFs typically originate from resident fibroblasts or precursor cells activated by tumor-derived signals. They exhibit high heterogeneity and secrete diverse cytokines, growth factors, and ECM components, collectively remodeling the TME to facilitate tumor development. CAFs significantly modulate tumor behavior (123–125). Their functional plasticity and diversity not only promote tumor progression but also represent potential therapeutic targets. SeRNA-seq has revealed substantial CAFs heterogeneity, identifying multiple transcriptionally distinct CAF subtypes within the TME (**Table 3**) (126–128).

### 4.1 Spatially resolved roles of CAFs in the TME

CAFs interact extensively with immune and tumor cells within the TME, significantly influencing tumor progression (129). A 2023 spatial transcriptomics study of 16 glioblastoma (GBM) patient samples demonstrated spatial proximity between CAFs, mesenchymal GBM stem cells, endothelial cells, and M2-like macrophages (130). Beyond immune modulation, CAFs shape the GBM vascular microenvironment (131). CAF-induced hypertrophic remodeling of tumor vasculature potentially underlies GBM resistance. ST revealed CAFs were preferentially localized in perivascular niches along with glioblastoma stem cells (GSCs), suggesting the interactions contributing to therapeutic resistance. These findings highlight CAF–GSC interactions as critical targets for therapeutic intervention in GBM (132).

### 4.2 CAFs in tumor metastasis

A 2022 study identified two major CAFs subtypes—iCAFs and myCAFs—in esophageal squamous cell carcinoma (ESCC), revealing the heterogeneity (133). Integrative scRNA-seq and ST analyses demonstrated the epithelial cells primarily localized in cancerous regions, whereas iCAFs were predominantly enriched in surrounding stroma. In contrast, myCAFs showed no distinct spatial preference. This spatial distribution suggested a pivotal role for iCAFs in tumor progression and metastasis.

### 4.3 CAFs remodeling in response to neoadjuvant chemotherapy

Neoadjuvant chemotherapy (NACT), administered before surgery or radiotherapy, reduces the tumor burden, enhances resection success, and eradicates micrometastases. Emerging evidence indicates NACT significantly reshapes CAF composition and function, influencing therapeutic outcomes. In rectal cancer, scRNA-seq demonstrated a distinct reorganization of CAFs following NACT, particularly characterized by an increase in myofibroblast populations after treatment. Elevated myCAFs facilitated ECM remodeling and immunosuppression, correlating with the poor prognosis (134, 135). However, the relationship between CAFs heterogeneity and NACT response remains incompletely characterized (136–139). Integrative scRNA-seq and ST analyses have begun to shed light on how NACT-induced remodeling affects therapeutic efficacy. In 2023, using combined scRNA-seq and STs, Qin et al. (140) identified a novel CAFs subpopulation termed positive-response–associated CAFs (pCAFs), which promoted anti-tumor immunity through spatial recruitment and immune cell interactions. Similar CAFs remodeling patterns were observed in pancreatic ductal adenocarcinoma (PDAC) (141). These findings indicate that NACT profoundly remodels both cancer cells and fibroblasts, leading to the formation of distinct immunological and stromal niches.

Collectively, these insights highlight the therapeutic potential of modulating specific CAFs subsets. Potential strategies include promoting immune-supportive pCAF differentiation, inhibiting tumor-promoting nCAF subpopulations, or targeting specific cytokines and ECM components driving therapy resistance. Nevertheless, the mechanisms underlying CAF heterogeneity are not yet fully understood. Systematic characterization of CAFs subsets and their context-specific functions will be essential for uncovering novel therapeutic targets.

## 5 Challenges and perspectives

Although scRNA-seq and ST have significantly enhanced our understanding of tumor biology, several challenges remain to be addressed (142). Tumors exhibit extensive somatic genetic heterogeneity (143), and their pathogenesis involves intricate regulatory mechanisms across multiple omics dimensions, including transcriptomics, epigenomics, proteomics, and metabolomics (144). With the rapid advancement of single-cell multi-omics technologies, research has increasingly transitioned from single-omics analyses to integrated approaches combining transcriptomic, genomic, epigenomic, and proteomic data. Such integrated multi-omics strategies have already provided valuable insights into several malignancies, including colorectal cancer (CRC) (145), lung cancer (146), and prostate cancer (147). The combination of single-cell multi-omics with ST is anticipated to offer a more comprehensive and spatially resolved understanding of tumor heterogeneity at single-cell resolution.

However, despite these technological advancements, clinical translation remains challenging. Several practical barriers remain for clinical transformation: (i) Cost-benefit trade-off: these technologies are currently expensive and have long experimental cycles; (ii) High requirements of infrastructure and data analysis capabilities; (iii) Lack of regulations and standardization. This requires collaborative efforts from regulators, industry, and academia (148, 149).

## 6 Outstanding questions

Achieving true single-cell resolution in spatial transcriptomics technologies and the associated computational challenges in analyzing such high-dimensional data. The necessary next step of integrating spatial multi-omics data, particularly spatial proteomics and metabolomics, to build a more comprehensive functional understanding of the tumor microenvironment. The urgent need for standardizing and validating analytical pipelines to ensure robustness, reproducibility, and ultimately, their successful translation into clinical settings for diagnostics and therapeutic decision-making.

## 7 Conclusion

In conclusion, the integration of single-cell and spatial transcriptomics technologies has fundamentally expanded our understanding of tumor heterogeneity and microenvironmental organization. However, to translate these insights into clinical impact, future work must focus on three critical frontiers. First, the integration of single-cell and spatial transcriptomics will be essential to move beyond transcriptional data and achieve a functional, multi-layered understanding of cellular phenotypes and interactions within their native context. Second, the prospective clinical validation of spatial biomarkers is urgently needed to establish their utility in patient stratification, prognosis, and therapy guidance. This will require rigorous standardization of analytical and reporting protocols to ensure reproducibility across platforms and cohorts. Finally, the development of advanced computational frameworks capable of unifying multi-omic spatial data—and ultimately enabling real-time mapping—will be crucial for informing diagnostic and even intraoperative decisions. With sustained development, these integrative approaches hold substantial promise for enhancing cancer diagnostics, guiding precision therapeutic strategies, and ultimately improving clinical outcomes for patients.
